# Supplementary material for: A multi-layer perceptron neural network for varied conditional attributes in tabular dispersed data
Source: PLoS One. 2024 Dec 2;19(12):e0311041. doi: 10.1371/journal.pone.0311041 (PMC11611123; doi:10.1371/journal.pone.0311041)
Supplement: Attachment — Submitted filename: Response to Reviewers.pdf [file pone.0311041.s001.pdf]

Dear Reviewers,

Thank you very much for your thoughtful review and valuable feedback on our article. We sincerely appreciate the time and effort you invested in providing such detailed insights. Your comments have been immensely helpful in refining our work to meet higher standards of clarity and depth.

Below, I address each of the points raised by the reviewers:

Reviewer #1:

Thank you for your insightful and valuable comments. We appreciate the time and effort you have taken to review our paper. Your feedback is highly constructive and will significantly enhance the quality of our work. Below are our responses to your specific points:

1. The introduction is very vague. It is full of citation without proper justification. The author should discuss in detail each citation that contributed to the work.

Response: The introduction is revised to provide a detailed discussion of each citation and how it contributes to the work. This will include a clearer explanation of the context and significance of the referenced studies. Changes are shown in blue.

2. The discussion of recent MLP should be include in Section 2.

Response: Section 2 is expanded to include a review of recent works on MLP. This will ensure the discussion is up-to-date and relevant. Changes are shown in blue.

3. Inclusion of Recent Works (2022-2023) in Sections 1 and 2:

Response: Additional recent studies from 2022-2023 is incorporated into both Section 1 and Section 2 to enhance the literature review and provide a more current perspective. A total of 13 new works have been added.

4. The author should include a schematic diagram of the MLP used in the proposed model.

Response: A schematic diagram of the proposed MLP model is included on page 7. The suggested paper "Weighted Random k Satisfiability for k = 1,2 (r2SAT) in Discrete Hopfield Neural Network" is cited for reference.

5. The author should analyze more number of dataset.

Response: Additional datasets are included in the analysis to validate the robustness and generalizability of the proposed model. Ten additional dispersed data have been added.

6. Fig 9 is awkwardly placed in Page 37.

Response: The placement of Fig 9 is adjusted to ensure it is appropriately positioned within the text.

7. I strongly suggest the author avoid short paragraphs throughout the paper.

Response: The manuscript is revised to avoid short paragraphs, ensuring a smoother flow of information throughout the paper.

8. The experimental setup of the conducted simulation is too simple and vague. I suggest the author include a section on Experimental Setup which include the following:

Response: Experimental Setup was carefully adjusted to the reviewer's suggestion. The division used was as indicated:

Simulation platform

Data set

Parameter Assignments

Formulations of the Performance Metrics

Reproducibility of the proposed model

Baseline methods

The papers are referenced.

9. Can the author justify how the parameters in MLP were chosen? If possible, can the author conduct a parameter tuning process?

Response: The parameter tuning process is detailed, including the criteria and methods used to choose the parameters for the MLP in Parameter Assignments Section.

10. I suggest the author conduct more comparative analysis with other Artificial Neural Network.

Response: For now, when the article is already quite long, we leave this part to work on for the future. Given that we can now very clearly see in experiments the advantage of the proposed method.

11. The author should consider Friedman test/other non-parametric test to validate the superiority of the proposed model as compared to other algorithms/baselines. Kindly refer/cite the following papers on how to do the Friedman test

Response: The Friedman test and other non-parametric tests are applied to validate the superiority of the proposed model. The recommended papers are cited for methodology. Pages 42-46.

12. Can the author justify the complexity value of the proposed model?

Response: A section justifying the complexity value of the proposed model is added, explaining the computational requirements and efficiency. On page 46-47 (shown in blue), such an analysis is presented in an additional paragraph

Reviewer #2:

Thank you for your valuable and constructive feedback on our manuscript. We address each of your comments as follows:

1. Elaborate on how data is collected and organized in local tables. Clarifying the selection criteria for objects and attributes in these tables will help readers understand the foundation of your local models.

Response: The method of data collection and organization in local tables is elaborated, including the selection criteria for objects and attributes (in Section Data set in blue).

2. Provide more detailed explanations and methodological steps for the development of local models, including how artificial objects are integrated.

Response: Detailed methodological steps for developing local models, including the integration of artificial objects, is provided on page 7 in blue also Figure 2 was added.

3. Expand on the weighted techniques used for aggregating local models. Detail the criteria for weighting and how they influence the final model performance.

Response: The criteria and techniques for weighting local models is discussed in detail, explaining their impact on final model performance on page 10-11 in blue also Figure 3 was added.

4. Describe the retraining process with global objects more thoroughly. Specify how this step integrates with the overall model development and its impact on model accuracy.

Response: The retraining process with global objects is described, highlighting its integration with overall model development and its effect on accuracy, at the beginning of Section Re-training the global MLP network with a sample of data, page 10-11 (in blue).

5. While you mention outperforming existing approaches, additional details about these methods and why your approach is superior would be valuable. Include a more in-depth comparative analysis or case studies.

Response: Additional details on existing methods and a deeper comparative analysis is included to substantiate the superiority of the proposed approach - last paragraph of page 46.

6. Provide more robust statistical evidence to support the claims of superiority in F1-score and precision. This could include confidence intervals, p-values, or other statistical tests.

Response: Robust statistical evidence, including confidence intervals, p-values, and other statistical tests, are provided to support claims of performance improvements. Pages 41-46.

7. Discuss the practical applications of your approach. How can it be implemented in real-world scenarios, and what are the potential challenges or limitations?

Response: The practical applications of the approach and potential challenges or limitations are discussed. This discussion was added on page 45-46.

8. The literature review section could effectively start by discussing the profound influence of artificial intelligence (AI) across multiple sectors.

Response: The first paragraph of the Related work section was added as indicated.

Reviewer #3:

Thank you for your valuable comments and insightful feedback. We appreciate your careful review of our paper and have addressed your concerns as follows:

1. More details (2 - 3 extra statements) should be added to the abstract highlighting the importance, and abit of detailed resuls, if possible.

Response: The abstract is revised to include more details on the importance and key results of the study. Changes are marked in blue.

2. How this model avoided overfitting, detailed answer of how the authors ensure avoid overfitting must include checking the performance for training and testing performance, at which point the model start overfitting.

Response: A detailed explanation of the measures taken to avoid overfitting, including performance checks on training and testing sets, are provided. Last paragraph of Section Re-training the global MLP network with a sample of data, page 11.

3. AUCROC must be plotted for the model and the comparison methods.

Response: AUC-ROC plots for the model and comparison methods are included to visually demonstrate performance. Pages 48-49 and description below.

4. More about the hyper-parameter and how they were obtained/optimized.

Response: Details on hyper-parameter optimization and the methods used to obtain them are included in Parameter Assignments Section.

5. The conclusion or discussion may detailed the limitations of the model.

Response: The conclusion is expanded to discuss the limitations of the model in detail. Page 50 in blue.

Reviewer #4:

Thank you for your valuable comments.

Response: The overall language of the paper, especially the introduction, is reviewed and improved for clarity and readability.

We appreciate the constructive feedback from all reviewers. The manuscript is revised accordingly to address each of these points comprehensively. These changes aim to enhance the clarity, depth, and overall quality of the paper.

We hope that these revisions meet your expectations and enhance the clarity and robustness of our paper.

Thank you once again for your valuable feedback.

Sincerely,
Małgorzata Przybyła-Kasperek
Kwabena Marfo
